# Supplementary material for: Intrinsic and induced quantum quenches for enhancing qubit-based quantum noise spectroscopy
Source: Nat Commun. 2021 Nov 11;12:6528. doi: 10.1038/s41467-021-26868-7 (PMC8586144; doi:10.1038/s41467-021-26868-7)
Supplement: Supplementary file 1 — Supplementary information. [file 41467_2021_26868_MOESM1_ESM.pdf]

Supplementary Information:
Intrinsic and induced quantum quenches for enhancing qubit-based quantum noise spectroscopy

Yu-Xin Wang[1] and Aashish A. Clerk[1]

[1] *Pritzker School of Molecular Engineering, University of Chicago, 5640 South Ellis Avenue, Chicago, Illinois 60637, U.S.A.*

Contents

Supplementary Note 1. Quench phase shift in comparison to relaxometry-based techniques to measure response 1

Supplementary Note 2. Asymptotic analysis on qubit dephasing and quench-induced phase shift for environments with power-law noise spectra and response functions 1

Supplementary Note 3. Alternative derivation of quench phase shift in Ohmic environments 3

Supplementary Note 4. Discussion on the use of Hahn echo versus Ramsey coherence times in quench-enhanced QNS for Ohmic bath thermometry 4

Supplementary Note 5. Case study: Quench phase shift generated by electromagnetic environment due to a driven damped cavity mode 5

Supplementary Note 6. General strategy for reconstructing the environmental spectral function in a generic frequency range using time-dependent quench functions 7

References 9

Supplementary Note 1. Quench phase shift in comparison to relaxometry-based techniques to measure response

In the main text, we have focused on the specific type of quantum noise spectroscopy measurements where a sensor qubit is coupled to its environment via pure-dephasing-type interactions. Within this setting, we have shown the quench phase shift (QPS) lets one probe the response properties, or spectral function of the environment, which would be otherwise inaccessible using standard dephasing-based noise spectroscopy.

Interestingly, in principle one can extract similar information about the imaginary part of response function $\mathrm{Im}G^R_{\xi\xi}[\omega]$, or equivalently the spectral function, using an extended version of standard T_1 relaxometry experiments. Conventional T_1-type experiments specifically probes transversely coupled bath fields (e.g., via $\hat{H}_{\rm int} = \hat{\sigma}_x \otimes \hat{\xi}$), which induces transitions between the qubit levels. In this setting, typically one would measure the qubit population decay rate $\Gamma_{\rm tot} \equiv 1/T_1$ [1], which corresponds to the sum of qubit relaxation and excitation rates. A straightforward calculation based on Fermi's Golden rule can then relate $\Gamma_{\rm tot}$ to the symmetrized noise spectral density (NSD) via $\Gamma_{\rm tot} = 2\bar{S}[\Omega]$, whereas the difference between relaxation and excitation rates corresponds to the response function $\mathrm{Im}G^R_{\xi\xi}[\Omega]$ (see the following paragraph for more detail). While the bath NSD can be directly inferred from T_1-decay rate $\Gamma_{\rm tot}$, to further probe response function $\mathrm{Im}G^R_{\xi\xi}[\omega]$ one would also need to measure the qubit steady state population $\langle\hat{\sigma}_z\rangle_{\rm ss}$: the latter measurement is not a part of standard T_1 relaxometry [2, 3]. In comparison, the QPS in Eq. (17) is readily accessible in standard T_2-type measurements, and as we show in the main text, arguably offers a more direct knob to probe the response properties of longitudinal bath fields. We also note that standard T_1-type experiments are not sensitive to dephasing baths. Although in principle one can use the spin-locking technique (also known as $T_{1\rho}$ measurements) [2], i.e. continuously drive the qubit to measure longitudinal bath fields via relaxometry-based experiments, in practice the drive strength needs to be higher than the inhomogeneous linewidth of the qubit. Thus, the range of frequencies that can be probed using the spin-locking technique often does not correspond to the dominating dephasing source for the undriven qubit (see e.g. [4–6]). In contrast, T_2-type experiments with QPS measurements are more suitable for probing low-frequency dephasing noise source.

We now concretely show the relation between qubit decay rate $\Gamma_{\rm tot}$ and the steady state population $\langle\hat{\sigma}_z\rangle_{\rm ss}$ to environmental properties. For the case of transverse coupling to the bath, it is more illuminating to represent bath properties in terms of the quantum noise spectra $S[\omega] \equiv \int_{-\infty}^{+\infty} dt e^{i\omega t}\langle\hat{\xi}(t)\hat{\xi}(0)\rangle$, so that the Fermi's Golden rule transition rates for qubit excitation and relaxation $\Gamma_\pm$ are given by $\Gamma_\pm = S[\mp\Omega]$ (Ω denotes qubit transition frequency; see Ref. [7] for a pedagogical introduction). One can use a few lines of algebra to show that the symmetrized and anti-symmetrized components of $S[\omega]$ are related to the bath NSD $\bar{S}[\omega] = (S[+\omega]+S[-\omega])/2$, and the imaginary part of response function $\mathrm{Im}G^R_{\xi\xi}[\omega] = (S[-\omega]-S[+\omega])/2$, respectively. The qubit population decay rate $\Gamma_{\rm tot}$ and the steady state population $\langle\hat{\sigma}_z\rangle_{\rm ss}$ can then be computed explicitly as $\Gamma_{\rm tot} = \Gamma_+ + \Gamma_- = 2\bar{S}[\Omega]$, and $\langle\hat{\sigma}_z\rangle_{\rm ss} = \mathrm{Im}G^R_{\xi\xi}[\Omega]/\bar{S}[\Omega]$. Thus, given both the qubit decay rate and steady state population simultaneously, we can use them to infer the response function [7–10].

Supplementary Note 2. Asymptotic analysis on qubit dephasing and quench-induced phase shift for environments with power-law noise spectra and response functions

In the main text, we present the asymptotic long-time behavior of qubit dephasing function and quench phase shift (QPS) in Eqs. (28) and (29), assuming that the bath noise spectral density (NSD) and density of states functions exhibit power-law dependence in the asymptotic low-frequency limit. For clarity, in this Supplementary Note, we provide a detailed derivation of the asymptotic results. We start with the general expressions for the dephasing function $\zeta(t_f)$ and QPS $\Phi_{\rm q}(t_f)$ under any spin-echo qubit control pulse, given by Eqs. (16) and (20) in the main text as

$$\zeta(t_f) = \int_{-\infty}^{+\infty} \frac{d\omega}{4\pi} |F[\omega]|^2 \bar{S}[\omega], \tag{1a}$$

$$\Phi_{\rm q}(t_f) = -\int_{-\infty}^{+\infty} \frac{d\omega}{\pi\omega} \mathrm{Re}F[\omega]\, \mathrm{Im}G^R_{\xi V}[\omega]. \tag{1b}$$

We also assume that the bath NSD $\bar{S}[\omega]$ and response functions $\mathrm{Im}G^R_{\xi V}[\omega]$ exhibit power-law dependence in the asymptotic low-frequency regime (see Eqs. (25) and (26) in the main text)

$$\bar{S}[\omega] \sim S_0\omega^p \quad (\omega \to 0^+), \tag{2a}$$

$$\mathrm{Im}G^R_{\xi V}[\omega] \sim -\frac{A_0}{2}\omega^s \quad (\omega \to 0^+). \tag{2b}$$

For convenience, we rewrite the bath NSD $\bar{S}[\omega]$ and response functions $\mathrm{Im}G^R_{\xi V}[\omega]$ in the full frequency range in terms of cutoff functions $\mu_A(x)$ $(A = S, G)$ as

$$\bar{S}[\omega] = S_0\omega^p \mu_S(\omega/\omega_c), \tag{3a}$$

$$\mathrm{Im}G^R_{\xi V}[\omega] = -\frac{A_0}{2}\omega^s \mu_G(\omega/\omega_c), \tag{3b}$$

where we introduce a UV cutoff frequency ω_c below which the asymptotic power-law function provides a good approximation for the exact function. By definition, the cutoff functions $\mu_A(x)$ satisfy following conditions

$$\mu_A(0) = 1, \quad \lim_{x\to\infty} \mu_A(x) = 0, \quad (A = S, G), \tag{4}$$

and we further assume both cutoff functions $\mu_A(x)$ $(A = S, G)$ corresponding to any physical bath are smooth near $x = 0$.

We now consider a generic qubit control pulse satisfying $F[0] = 0$, which consists of L instantaneous π-pulses at times $t = \alpha_\ell t_f$. Without loss of generality, we assume the coefficients α_ℓ $(\ell = 1, 2, \ldots, L)$ satisfy following conditions

$$\alpha_1 < \alpha_2 < \ldots < \alpha_L, \quad F[0] = 0 \Leftrightarrow 2\sum_{\ell=1}^{L}(-)^\ell\alpha_\ell + (-)^{L+1} = 0, \tag{5}$$

so that we can explicitly compute the filter function $F[\omega]$ as

$$F[\omega] = \int_0^{t_f} dt_1 F(t_1)e^{i\omega t_1} = \frac{2\sum_{\ell=1}^{L}(-)^{\ell-1}e^{i\alpha_\ell\omega t_f} - 1 + (-)^L e^{i\omega t_f}}{i\omega}. \tag{6}$$

Substituting above equation into Supplementary Eqs. (1) and noting that the integrands are even functions of frequency, we obtain

$$\begin{aligned}\zeta(t_f) &= \frac{S_0}{2\pi}\int_0^{+\infty}\omega^{p-2}\left|2\sum_{\ell=1}^{L}(-)^\ell e^{i\alpha_\ell\omega t_f} + 1 + (-)^{L+1}e^{i\omega t_f}\right|^2 \mu_S(\omega/\omega_c)\,d\omega \\ &= \frac{S_0}{2\pi}t_f^{1-p}\int_0^{+\infty}x^{p-2}\left|2\sum_{\ell=1}^{L}(-)^\ell e^{i\alpha_\ell x} + 1 + (-)^{L+1}e^{ix}\right|^2 \mu_S(x/\omega_c t_f)\,dx,\end{aligned} \tag{7a}$$

$$\begin{aligned}\Phi_q(t_f) &= \frac{A_0}{\pi}\int_0^{+\infty}\omega^{s-2}\left[2\sum_{\ell=1}^{L}(-)^{\ell-1}\sin\alpha_\ell\omega t_f + (-)^L\sin\omega t_f\right]\mu_G(\omega/\omega_c)\,d\omega \\ &= \frac{A_0}{\pi}t_f^{1-s}\int_0^{+\infty}x^{s-2}\left[2\sum_{\ell=1}^{L}(-)^{\ell-1}\sin\alpha_\ell x + (-)^L\sin x\right]\mu_G(x/\omega_c t_f)\,dx.\end{aligned} \tag{7b}$$

In the long-time limit $t_f \to +\infty$, the integrals above would tend asymptotically to universal limits that are independent of details about the physical cutoffs, if and only if the integrals when setting $\mu_A(x) \equiv 1$ $(A = S, G)$ are well defined. For this scenario, the asymptotic limits of dephasing function and phase shift functions can be derived as

$$-3 < p < 1: \ \zeta(t_f) \sim \mathcal{C}_\zeta(p)S_0 t_f^{1-p} \quad (t_f \to +\infty), \tag{8a}$$

$$-2 < s < 2: \Phi_q(t_f) \sim \mathcal{C}_\Phi(s)\frac{A_0}{2}t_f^{1-s} \quad (t_f \to +\infty), \tag{8b}$$

where the dimensionless coefficients $\mathcal{C}_\zeta(p)$ and $\mathcal{C}_\Phi(s)$ are determined by the spin-echo pulse parameters as

$$\mathcal{C}_\zeta(p) = \frac{\Gamma(p-1)}{\pi}\left\{4\sum_{\ell>\ell'}^{L}(-)^{\ell+\ell'}(\alpha_\ell - \alpha_{\ell'})^{1-p} + 2\sum_{\ell=1}^{L}(-)^\ell\left[(-)^{L+1}(1-\alpha_\ell)^{1-p} + \alpha_\ell^{1-p}\right] + (-)^{L+1}\right\}\sin\frac{p\pi}{2}, \tag{9a}$$

$$\mathcal{C}_\Phi(s) = \frac{\Gamma(s-1)}{\pi}\left[2\sum_{\ell=1}^{L}(-)^\ell\alpha_\ell^{1-s} + (-)^{L+1}\right]\cos\frac{s\pi}{2}, \tag{9b}$$

and $\Gamma(\cdot)$ is the gamma function. For Hahn echo, the control pulse parameters are $L=1$, $\alpha_1=\frac{1}{2}$, and substituting the parameters into equations above lets us obtain the coefficients $\mathcal{C}_{\zeta,\mathrm{H}}=\frac{1-2^{p+1}}{\pi}\Gamma(p-1)\sin\frac{p\pi}{2}$ and $\mathcal{C}_{\Phi,\mathrm{H}}=\frac{1-2^{s}}{\pi}\Gamma(s-1)\cos\frac{s\pi}{2}$ in the main text. Note that above equations are still well-defined if p,s are exact integers, where the gamma function in Supplementary Eqs. (9) alone might diverge: in this case, we could obtain the asymptotic coefficients by taking the continuous limit of Supplementary Eqs. (9) as the exponent approaches the corresponding integer value. The asymptotic limit of quench phase shift can be further simplified if the response function exponent take the value of 1, as

$$s=1:\lim_{t_f\to+\infty}\Phi_{\mathrm{q}}(t_f)=A_0/2. \tag{10}$$

For exponents beyond the range of validity specified in Supplementary Eqs. (8), the long-time behavior of the dephasing function (phase shift) may not have a well-defined asymptotic limit, or the asymptotic behavior would depend on details of the low- or high-frequency cutoff of the bath NSD (response function). To illustrate this, we discuss a concrete example where the long-time phase shift dynamics explicitly depends on details of the cutoff. We compare the Hahn echo phase shift dynamics for response function $\mathrm{Im}G^R_{\xi V}[\omega]=-(A_0/2)\omega^s\mu_G(\omega/\omega_\mathrm{c})$ with exponent $s=5/2$, and two different UV cutoff functions: exponential cutoff with $\mu_{G,\exp}(x)=e^{-x}$, and step-function cutoff with $\mu_{G,\mathrm{sp}}(x)=\Theta(1-x)$, where $\Theta(\cdot)$ is the Heaviside step function. The quench phase shift is generally given by Supplementary Eq. (7b), which for Hahn echo can be computed analytically to yield

$$\Phi_{\mathrm{q,exp}}(t_f)=\frac{4A_0}{\pi}\int_0^{+\infty}\omega^{\frac{1}{2}}e^{-\frac{\omega}{\omega_\mathrm{c}}}\sin\frac{\omega t_f}{2}\sin^2\frac{\omega t_f}{4}d\omega \tag{11}$$

$$=\frac{A_0}{2\sqrt{\pi}}t_f^{-\frac{3}{2}}\left[2^{\frac{3}{2}}e^{i\frac{\pi}{4}}\left(1+\frac{2i}{\omega_\mathrm{c}t_f}\right)^{-\frac{3}{2}}-2^{-1}e^{i\frac{\pi}{4}}\left(1+\frac{i}{\omega_\mathrm{c}t_f}\right)^{-\frac{3}{2}}+\mathrm{c.c.}\right], \tag{12}$$

$$\Phi_{\mathrm{q,sp}}(t_f)=\frac{4A_0}{\pi}\int_0^{\omega_\mathrm{c}}\omega^{\frac{1}{2}}\sin\frac{\omega t_f}{2}\sin^2\frac{\omega t_f}{4}d\omega \tag{13}$$

$$=\frac{8A_0}{\pi}\left[\frac{\sqrt{\omega_\mathrm{c}}}{t_f}\sin^4\frac{\omega_\mathrm{c}t_f}{4}-\frac{\sqrt{\omega_\mathrm{c}}}{2t_f}\int_0^1 x^{-\frac{1}{2}}\sin^4\frac{x\omega_\mathrm{c}t_f}{4}dx\right]. \tag{14}$$

While the asymptotic $t_f\gg\omega_\mathrm{c}^{-1}$ limit of Hahn echo phase shift $\Phi_{\mathrm{q,exp}}(t_f)$ assuming exponential cutoff agrees with the universal result in Supplementary Eq. (8b), it is straightforward to see that the phase shift dynamics with step-function cutoff does not have a well-defined asymptotic long-time limit, and does not agree with Supplementary Eq. (8b). Although the oscillatory behavior of the first term in the square bracket in Supplementary Eq. (14) is typical when we have response functions with a step-function cutoff (e.g. see the dashed blue curve in Fig. 5c in the main text, depicting the QPS for Ohmic bath spectral function with a step-function UV cutoff), for exponents within the range of validity of Supplementary Eq. (14) such oscillations are negligible in the asymptotic long-time limit. However, as shown in Supplementary Eq. (14), for exponents outside this range the oscillatory contribution is important even in the long-time limit. Generally, for bath NSD (response function) of the form given by Supplementary Eqs. (2) with exponent $p\geq 1$ ($s\geq 2$), the long-time behavior of the dephasing function $\zeta(t_f)$ (quench phase shift $\Phi_\mathrm{q}(t_f)$) depend on the detail of the UV cutoff of the spectrum. Similarly, for exponents $p\leq -3$ ($s\leq -2$) below the regime of validity in Supplementary Eq. (8), the corresponding long-time behavior would depend on the low-frequency cutoff.

Supplementary Note 3. Alternative derivation of quench phase shift in Ohmic environments

As discussed in the main text, specifically for baths that exhibit Ohmic behavior (a flat NSD and a linear bath spectral function) in the asymptotic low-frequency limit, i.e., satisfying Supplementary Eq. (2) with $p=0$, $s=1$, the quench phase shift (QPS) under spin-echo or dynamical-decoupling control sequences tends to a constant in the long-time regime, as shown in Eq. (31) (see also Supplementary Eq. (10) in Supplementary Note 2). In this Supplementary Note, we provide an intuitive derivation of this result, which for a generic control sequence can be written as

$$\lim_{t_f\to+\infty}\Phi_\mathrm{q}(t_f)=\frac{F[0]}{2}\mathrm{Re}G^R_{\xi\xi}[\omega=0^+]-\frac{1}{2}\left.\frac{d\mathrm{Im}G^R_{\xi\xi}[\omega]}{d\omega}\right|_{\omega=0^+}, \tag{15}$$

where $F[0]\equiv\int_0^{t_f}F(t)dt$. We start with the general linear response formula for QPS, assuming quench operator $\hat{V}=\hat{\xi}/2$, quench function $\eta(t)=\Theta(t)\Theta(t_f-t)$, and a generic filter function, which in the time domain is given by

(see Eq. (77) in the main text)

$$\Phi_{\rm q}(t_f) = \frac{1}{2}\int_0^{t_f} dt_1 F(t_1)\int_0^{t_1} dt_2 G^R_{\xi\xi}(t_1-t_2). \tag{16}$$

We can rewrite the expression on the right hand side using integration by parts as

$$\Phi_{\rm q}(t_f) = \frac{F[0]}{2}\int_0^{t_f} dt_1 G^R_{\xi\xi}(t_1) - \frac{1}{2}\int_0^{t_f} dt_1 G^R_{\xi\xi}(t_1)\int_0^{t_1} dt_2 F(t_2). \tag{17}$$

The first term on the RHS can be viewed as the net phase shift due to a constant qubit frequency shift $\int_0^{t_f} dt G^R_{\xi\xi}(t)$ accumulated during the time evolution, whereas the second term accounts for a residual phase correction due to the fact that the quench-induced frequency shift to the qubit is time dependent. For Ohmic baths whose response functions exhibit linear dependence in the asymptotic low-frequency regime, it is straightforward to show that the asymptotic long-time behaviors of these two terms are given by

$$\lim_{t_f\to+\infty}\frac{F[0]}{2}\int_0^{t_f} dt G^R_{\xi\xi}(t) = \frac{F[0]}{2}\int_0^{+\infty} G^R_{\xi\xi}(t)dt = \frac{F[0]}{2}\mathrm{Re}G^R_{\xi\xi}[\omega=0^+], \tag{18}$$

$$-\frac{1}{2}\lim_{t_f\to+\infty}\int_0^{t_f} dt_1 G^R_{\xi\xi}(t_1)\int_0^{t_1} dt_2 F(t_2) = -\frac{1}{2}\int_0^{+\infty} tG^R_{\xi\xi}(t)dt = -\frac{1}{2}\left.\frac{d\mathrm{Im}G^R_{\xi\xi}[\omega]}{d\omega}\right|_{\omega=0^+}. \tag{19}$$

Thus, the asymptotic long-time behavior of QPS with Ohmic baths can be viewed as the sum of phase shift due to a static frequency shift in the long-time limit, which is proportional to $F[0]$, and a residual phase correction. Noting that the bath spectral function $\mathcal{J}[\omega]$ is related to the response function via $\mathcal{J}[\omega] = -\frac{1}{\pi}\mathrm{Im}\, G^R_{\xi\xi}[\omega]$ (see also Eq. (22) in the main text), we have

$$\begin{aligned}\lim_{t_f\to+\infty}\Phi_{\rm q}(t_f) &= \frac{F[0]}{2}\mathrm{Re}G^R_{\xi\xi}[\omega=0^+] - \frac{1}{2}\left.\frac{d\mathrm{Im}G^R_{\xi\xi}[\omega]}{d\omega}\right|_{\omega=0^+} \\ &= \frac{F[0]}{2}\mathrm{Re}G^R_{\xi\xi}[\omega=0^+] + \frac{\pi}{2}\left.\frac{d\mathcal{J}[\omega]}{d\omega}\right|_{\omega=0^+}.\end{aligned} \tag{20}$$

Specifically for dynamical-decoupling-type control pulses with $F[0]=0$, the first term would vanish, and we recover Eq. (31) in the main text. As a result, for approximately Ohmic baths with spectral function satisfying $\mathcal{J}[\omega]\sim\omega$ at low frequencies, the asymptotic behavior of QPS under spin-echo control pulses in the long time $t_f\to\infty$ regime is universal (i.e., it only depends on the asymptotic linear dependence of the spectral function), and is independent of the specific UV cutoff of the response function and details of the qubit control sequence.

Supplementary Note 4. Discussion on the use of Hahn echo versus Ramsey coherence times in quench-enhanced QNS for Ohmic bath thermometry

In the main text, we show that our quench-enhanced T_2-style quantum noise spectroscopy offers a direct route (i.e. without any curve fitting) to estimating the temperature of any baths exhibiting Ohmic behavior in the asymptotic low-frequency limit, i.e. $\bar{S}[\omega]\sim$ const. and $\mathcal{J}[\omega]\sim\omega$ as $\omega\to 0^+$. However, realistic systems may also experience a large amount of quasistatic noise, leading to deviations from perfect Ohmic behavior at infinitesimal frequencies. In this Supplementary Note, we discuss how our thermometry protocol also works in the presence of such quasistatic noise.

Recall that our protocol can be summarized in Eq. (32) in the main text, where we can extract bath temperature T from the quench phase shift $\Phi_{\rm q}(t_f)$ and low-frequency noise spectral density $\bar{S}[0]$, via the following relation

$$T = \frac{\bar{S}[0]}{4k_B}\left[\lim_{t_f\to+\infty}\Phi_{\rm q}(t_f)\right]^{-1}. \tag{21}$$

Note that this result assumes the NSD is flat in the low-frequency limit, in which case the qubit Ramsey and Hahn-echo coherence times are necessarily identical. Here we stress that even in the circumstances where the qubit Hahn-echo time T_2 differs from the Ramsey coherence time $T_{\rm FID}$, a modified version of Supplementary Eq. (21) is still applicable, as long as the slow noise disrupting Ohmic NSD behavior emerges at a much lower frequency scale compared to

the Ohmic regime. More specifically, this means the NSD $\bar{S}[\omega]$ and the spectral function $\mathcal{J}[\omega]$ has a low-frequency cutoff $\omega_{\rm ir}$, below which the Ohmic behavior $\bar{S}[\omega] \sim$ const. and $\mathcal{J}[\omega] \sim \omega$ breaks down. As mentioned, this includes the common physical situations, where the environment also has a large amount of quasistatic noise, which can be described as an additional delta function peak in the NSD.

It then follows that, our thermometry protocol is applicable to baths with asymptotic low-frequency Ohmic behavior, which may exhibit a high- as well as a low-frequency cutoff. For this more general scenario, we should use asymptotic low-frequency NSD $\lim_{\omega\to 0}\bar{S}[\omega] = 2/T_2$, instead of strictly zero-frequency noise $\bar{S}[0] = 2/T_{\rm FID}$ in Supplementary Eq. (21). This justifies the use of Hahn-echo coherence time T_2 in the main text.

Supplementary Note 5. Case study: Quench phase shift generated by electromagnetic environment due to a driven damped cavity mode

In the main text, we have considered a quantum bath whose spectral function is asymptotically Ohmic in the low-frequency limit (i.e. $\bar{S}[\omega] \sim$ const. and $\mathcal{J}[\omega] \sim \omega$ as $\omega \to 0^+$) and also exhibits a Lorentzian peak at a finite frequency; the Hahn-echo quench phase shift (QPS) dynamics due to this bath is illustrated in Fig. 5b. As mentioned in the main text, such spectral function can describe dephasing environments generated by a driven damped electromagnetic (EM) cavity. In this Supplementary Note, we provide a detailed discussion on the corresponding physical system.

Consider a qubit dispersively coupled to a driven damped bosonic mode b (resonance frequency $\omega_{\rm c}$, decay rate κ) via the Hamiltonian

$$\hat{H}_{\rm int} = \frac{1}{2}\hat{\sigma}_z \otimes \hat{\xi}, \quad \hat{\xi} = \lambda \hat{b}^\dagger \hat{b}. \tag{22}$$

For instance, we may have a superconducting transmon qubit coupled to a microwave cavity mode; the photon shot noise fluctuations due to the cavity mode then induce qubit frequency shift and dephasing during time evolution. Transforming to the interaction picture defined by the free qubit Hamiltonian $\Omega\hat{\sigma}_z/2$, as well as frame rotating at the drive frequency $\omega_{\rm dr}$ of the cavity mode, the dynamics of the total system can be described by the quantum master equation as follows

$$\dot{\hat{\rho}} = -i[\hat{H}_0 + \hat{H}_{\rm int} + \hat{H}_{\rm dr}, \hat{\rho}] + \kappa(\bar{n}_{\rm th}+1)\mathcal{D}[\hat{b}]\hat{\rho} + \kappa\bar{n}_{\rm th}\mathcal{D}[\hat{b}^\dagger]\hat{\rho}, \tag{23}$$

where $\bar{n}_{\rm th}$ is the thermal photon number. For simplicity, we assume zero temperature ($\bar{n}_{\rm th} = 0$) hereafter, but we stress that our approach also applies to the case with finite $\bar{n}_{\rm th}$. In the above equation, $\hat{H}_0$ and $\hat{H}_{\rm dr}$ are rotating-frame Hamiltonians accounting for free cavity dynamics and the linear cavity drive, respectively, as

$$\hat{H}_0 = -\Delta \hat{b}^\dagger \hat{b}, \quad \hat{H}_{\rm dr}(t) = y(t) f_{\rm dr} \hat{b}^\dagger + \text{H.c.}, \tag{24}$$

where $\Delta \equiv \omega_{\rm dr} - \omega_{\rm c}$ denotes the cavity detuning, and $f_{\rm dr}$ is the drive strength. We introduce a dimensionless envelope function $y(t)$ to encode possible time dependence of the drive. For the purpose of our discussion, we can assume the cavity drive is switched on at some earlier time before the start of the Hahn-echo protocol, so that we have $y(t) = 1$ during the protocol ($0 < t < t_f$). For convenience, we define the stationary intracavity driven photon number in the absence of the qubit (i.e., setting $\lambda = 0$ in Supplementary Eq. (23)) as $\bar{n}_{\rm dr}$, so that we have (here $\langle\cdot\rangle$ denotes stationary state expectation values of bath operator)

$$\bar{n}_{\rm dr} = |\beta_{\rm dr}|^2, \quad \beta_{\rm dr} = \langle \hat{b} \rangle = \frac{f_{\rm dr}}{\Delta + i\frac{\kappa}{2}}. \tag{25}$$

In this specific setup, the photon shot noise coupled to the qubit is generally non-Gaussian [11]. Thus, in order to apply our results in the main text (see also Supplementary Eqs. (1)) to describe the photonic environment, we first need to ensure non-Gaussian effects are small. Without loss of generality, we focus on the mean-field regime where the Gaussian approximation is well justified, i.e. we have approximately

$$\delta\hat{\xi} \equiv \lambda \hat{b}^\dagger \hat{b} - \lambda \langle \hat{b}^\dagger \hat{b} \rangle \simeq \lambda \beta_{\rm dr} \delta\hat{b}^\dagger + \text{H.c.}, \tag{26}$$

which holds if we require parameters to satisfy following conditions

$$\bar{n}_{\rm dr} > 1, \quad \sqrt{\bar{n}_{\rm dr}}\frac{\lambda}{\kappa} \lesssim 1. \tag{27}$$

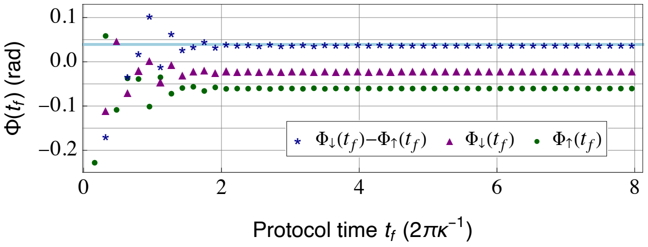

Supplementary Fig. 1: Numerically simulated Hahn-echo qubit phase shift and the quench phase shift dynamics, corresponding to the photonic environment generated by a driven, damped cavity mode. The purple triangles (green circles) depict qubit phase shifts $\Phi_{\downarrow}(t_f)$ ($\Phi_{\uparrow}(t_f)$) using initial qubit state $|\downarrow\rangle$ ($|\uparrow\rangle$), which are computed numerically from solving the qubit-cavity master equation in Supplementary Eq. (23). The difference between the two qubit phase shifts $[\Phi_{\downarrow}(t_f) - \Phi_{\uparrow}(t_f)]$ (dark blue asterisks) in the long-time limit can be well described by the asymptotic expression based on quench phase shift in Supplementary Eq. (35), as expected. Parameters: $\Delta/\kappa = 5$, $\lambda/\kappa = 0.5$, $\bar{n}_{\rm dr} = 10$, and $\bar{n}_{\rm th} = 0$.

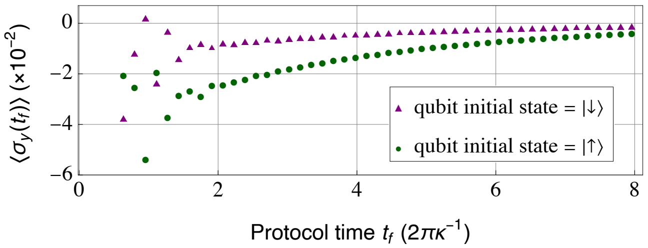

Supplementary Fig. 2: Numerically simulated Hahn-echo qubit coherence function $\langle\hat{\sigma}_y(t_f)\rangle$ corresponding to the photonic environment generated by a driven damped cavity mode. The purple triangles (green circles) depict qubit Hahn-echo coherence function $\langle\hat{\sigma}_y(t_f)\rangle$ using initial qubit state $|\downarrow\rangle$ ($|\uparrow\rangle$), which are computed numerically from solving the qubit-cavity master equation in Supplementary Eq. (23). Note that data points with small protocol times ($t_f < \pi/\kappa$) fall out of range of the figure and are not shown here; however, the short-protocol-time behavior is determined by the high-frequency components of the spectral function, and does not affect our conclusion. The parameters are the same as in Supplementary Fig. 1.

Substituting the mean field approximation (Supplementary Eq. (26)) into definition of symmetrized noise spectral density $\bar{S}[\omega]$ (see Eq. (14) in the main text)

$$\bar{S}[\omega] \equiv \frac{1}{2}\int_{-\infty}^{+\infty} dt e^{i\omega t}\langle\{\delta\hat{\xi}(t), \delta\hat{\xi}(0)\}\rangle, \tag{28}$$

and making use of solution to the master equation in Supplementary Eq. (23), we can straightforwardly obtain the NSD as

$$\bar{S}[\omega] = \bar{n}_{\rm dr}\lambda^2\left[\frac{\frac{\kappa}{2}}{\left(\frac{\kappa}{2}\right)^2 + (\Delta+\omega)^2} + \frac{\frac{\kappa}{2}}{\left(\frac{\kappa}{2}\right)^2 + (\Delta-\omega)^2}\right]. \tag{29}$$

Similarly we can derive the bath spectral function $\mathcal{J}[\omega]$ as

$$\mathcal{J}[\omega] = -\frac{1}{\pi}{\rm Im}G^R_{\xi\xi}[\omega] = \frac{\bar{n}_{\rm dr}\lambda^2}{\pi}\left[\frac{\frac{\kappa}{2}}{\left(\frac{\kappa}{2}\right)^2 + (\Delta-\omega)^2} - \frac{\frac{\kappa}{2}}{\left(\frac{\kappa}{2}\right)^2 + (\Delta+\omega)^2}\right]. \tag{30}$$

Note that we can rewrite the spectral function in Supplementary Eq. (30) as

$$\mathcal{J}[\omega] = \omega\frac{\bar{n}_{\rm dr}\lambda^2}{\pi\Delta^2}\frac{\frac{2\kappa}{\Delta}}{\left[\left(\frac{\omega}{\Delta}+1\right)^2 + \left(\frac{\kappa}{2\Delta}\right)^2\right]\left[\left(\frac{\omega}{\Delta}-1\right)^2 + \left(\frac{\kappa}{2\Delta}\right)^2\right]}, \tag{31}$$

which recovers the form of spectral function used to compute data shown in Fig. 5b, if we redefine centers of Lorentzian peaks as $\pm\Delta$. It is straigtforward to check that the photon shot noise exhibits Ohmic behavior in the asymptotic

low-frequency limit, i.e. we have

$$\bar{S}[\omega] \sim \bar{n}_{\rm dr}\lambda^2 \frac{\kappa}{\left(\frac{\kappa}{2}\right)^2 + \Delta^2} \quad (\omega \to 0^+), \tag{32a}$$

$$\mathcal{J}[\omega] \sim \frac{\bar{n}_{\rm dr}}{\pi} \frac{2\kappa\Delta\lambda^2}{\left[\left(\frac{\kappa}{2}\right)^2 + \Delta^2\right]^2}\omega \quad (\omega \to 0^+). \tag{32b}$$

Making use of results in Supplementary Eqs. (8), we thus obtain the asymptotic behavior of dephasing function $\zeta(t_f)$ and the quench phase shift $\Phi_{\rm q}(t_f)$

$$\zeta(t_f) \sim \bar{n}_{\rm dr} \frac{\lambda^2}{\left(\frac{\kappa}{2}\right)^2 + \Delta^2} \frac{\kappa t_f}{2} \quad (t_f \to +\infty), \tag{33a}$$

$$\Phi_{\rm q}(t_f) \sim \bar{n}_{\rm dr} \frac{\kappa\Delta\lambda^2}{\left[\left(\frac{\kappa}{2}\right)^2 + \Delta^2\right]^2} \quad (t_f \to +\infty). \tag{33b}$$

We now compare the predicted asymptotic results to exact dynamics from directly solving the master equation in Supplementary Eq. (23). We consider qubit dynamics corresponding to standard Hahn-echo protocol, where the qubit-bath system is initialized as follows: i) we first prepare the qubit in one of eigenstates, $|\uparrow\rangle$ or $|\downarrow\rangle$; ii) we then switch on cavity drive, and wait for long enough so that the cavity reaches a stationary state at the start ($t = 0$) of the Hahn echo protocol. Denoting the qubit phase shift corresponding to initial state $|\uparrow\rangle$ ($|\downarrow\rangle$) as $\Phi_\uparrow(t_f)$ ($\Phi_\downarrow(t_f)$), we thus have

$$\lim_{t_f \to +\infty} [\Phi_\downarrow(t_f) - \Phi_\uparrow(t_f)] = 2 \lim_{t_f \to +\infty} \Phi_{\rm q}(t_f) = \pi \left. \frac{d\mathcal{J}[\omega]}{d\omega} \right|_{\omega=0^+} \tag{34}$$

$$= \bar{n}_{\rm dr} \frac{2\kappa\Delta\lambda^2}{\left[\left(\frac{\kappa}{2}\right)^2 + \Delta^2\right]^2}. \tag{35}$$

Because the master equation conserves qubit polarization (i.e., $\hat{\sigma}_z$), and is quadratic in terms of bosonic mode operators, we can numerically simulate the exact qubit-cavity system dynamics efficiently (see [11] for details). Supplementary Fig. 1 illustrates the exact qubit phase shift dynamics corresponding to initial qubit state $|\downarrow\rangle$ and $|\uparrow\rangle$ (purple triangles and green circles, respectively) for the choice of parameters $\Delta/\kappa = 5$, $\lambda/\kappa = 0.5$, and $\bar{n}_{\rm dr} = 10$. As shown in Supplementary Fig. 1, the difference in qubit phase shifts $[\Phi_\downarrow(t_f) - \Phi_\uparrow(t_f)]$ (dark blue asterisks) in the long-time limit is in good agreement with the asymptotic result in Supplementary Eq. (35) (light blue line), verifying our approach. The corresponding Hahn-echo coherence functions are plotted in Supplementary Fig. 2, and can be readily measured using state-of-the-art implementations of superconducting qubits.

The results above on quench phase shift encode intriguing information about nature of the corresponding environment. Comparing the asymptotic results in Supplementary Eqs. (33) to the temperature estimation formula in Supplementary Eq. (21), the qubit quench phase shift dynamics, which can be detected by measuring $[\Phi_\downarrow(t_f) - \Phi_\uparrow(t_f)]$, indicates that low-frequency photon shot noise has a finite temperature. At first glance, this might seem surprising, as the system master equation in Supplementary Eq. (23) only includes a purely cooling dissipator when we assume zero thermal photon number ($\bar{n}_{\rm th} = 0$), and does not involve any explicit heating. We note that there is in fact no contradiction: the finite temperature of low-frequency fluctuations reflects the fact that Markovian dissipation actually corresponds to a non-equilibrium environment, and the quench approach provides a direct knob to probe this physics.

Supplementary Note 6. General strategy for reconstructing the environmental spectral function in a generic frequency range using time-dependent quench functions

In the main text, we discussed using sensor qubits based on a single nitrogen vacancy center in diamond to engineer a time-dependent quench (c.f. Fig. 6), and we discussed its application in reconstructing the bath spectral function for a specific type of control pulses. In this Supplementary Note, we discuss a general recipe to construct more general periodic control pulses, which lead to a powerful set of varying spectral filters that can be utilized to reconstruct the spectral function $\mathcal{J}[\omega]$ in a broad range of frequencies.

As discussed in the main text (see discussions following Eq. (33)), to illustrate the idea we focus on case where the quench operator is directly related to noise, with $\hat{V} = \hat{\xi}/2$. Without loss of generality, we also focus on periodic NV

control pulses, which are suitable for reconstructing the spectral function at finite target frequencies. Recall that the spin-1 structure of the NV lets us effectively realize a nontrivial quench function $\eta(t)$, in addition to the standard noise filter function. More specifically, we can apply a periodic sequence of NV control pulses (period T with $2M$ repetitions, $M \in \mathbb{Z}$), switching between the qubit subspaces $\{m_z = 0, m_z = +1\}$ and $\{m_z = 0, m_z = -1\}$ (see also Fig. 6 in the main text), to realize a periodic quench function as

$$\eta(t) = \sum_{m=0}^{M-1} \eta_0(t - 2mT; 2T), \tag{36}$$

where $\eta_0(t; 2T)$ denotes the base quench function, and satisfies the following relation

$$\eta_0(t; 2T) = \begin{cases} +1 \text{ or } -1, & 0 \leq t \leq 2T, \\ 0, & t < 0 \text{ or } t > 2T. \end{cases} \tag{37}$$

The structure of switching pulses also ensure that $\eta_0(t; 2T) = (-)^N \eta_0(t + T; 2T)$ for $0 < t < T$, where N is the total number of switching pulses per period T. For the example control pulse sequence depicted in Fig. 6, we have $N = 1$ and $\eta_0(t; 2T) = -\Theta(t)\Theta(T - t) + \Theta(t - T)\Theta(2T - t)$, where $\Theta(\cdot)$ denotes the Heaviside step function. Again introducing the total protocol time satisfying $t_f = 2MT$, we can straightforwardly rewrite Fourier transform of the quench function as

$$\eta[\omega] = e^{i(M-1)\frac{\omega t_f}{2M}} \frac{\sin \frac{\omega t_f}{2}}{\sin \frac{\omega t_f}{2M}} \eta_0[\omega; 2T], \tag{38}$$

$$\eta_0[\omega; 2T] \equiv \int_0^{2T} \eta_0(t; 2T) e^{i\omega t} dt. \tag{39}$$

For reasons that will become clear, we also assume a periodic sequence of standard qubit control π-pulses, with a same period T and total evolution time $t_f = 2MT$, so that we similarly have

$$F(t) = \sum_{m=0}^{M-1} F_0(t - 2mT; 2T), \tag{40}$$

$$F[\omega] = e^{i(M-1)\frac{\omega t_f}{2M}} \frac{\sin \frac{\omega t_f}{2}}{\sin \frac{\omega t_f}{2M}} F_0[\omega; 2T]. \tag{41}$$

Substituting Supplementary Eqs. (41) and (38) above into Eq. (17) in the main text, which described the general quench phase shift, and noting that $\hat{V} = \hat{\xi}/2$, we obtain

$$\begin{aligned} \Phi_{\mathrm{q}}(t_f) &= \int_{-\infty}^{+\infty} \frac{d\omega}{2\pi} F^*[\omega]\eta[\omega] G^R_{\xi V}[\omega] \\ &= \int_{-\infty}^{+\infty} \frac{d\omega}{4\pi} F^*[\omega]\eta[\omega] G^R_{\xi\xi}[\omega] \\ &= \int_{-\infty}^{+\infty} \frac{d\omega}{4\pi} \frac{\sin^2 \frac{\omega t_f}{2}}{\sin^2 \frac{\omega t_f}{2M}} F_0^*[\omega; 2T]\eta_0[\omega; 2T] G^R_{\xi\xi}[\omega]. \end{aligned} \tag{42}$$

We are now ready to present the recipe, or the necessary and sufficient conditions, to construct spectral filters that specifically probe the bath spectral function $\mathcal{J}[\omega]$ (see also Eq. (22) in the main text)

$$\mathcal{J}[\omega] = -\frac{1}{\pi} \mathrm{Im} G^R_{\xi\xi}[\omega]. \tag{43}$$

We essentially require that the base filter and quench functions exhibit the same periodicity, and satisfy the following conditions

- The base filter and quench functions must be mirror symmetric or anti-symmetric with respect to $t = T$, i.e., $F_0(t; 2T) = s_F F_0(2T - t; 2T)$, and $\eta_0(t; 2T) = s_\eta \eta_0(2T - t; 2T)$, where s_F, $s_\eta = \pm 1$.
- The base filter and quench functions exhibit opposite mirror symmetries with respect to $t = T$, i.e., $s_F = -s_\eta = +1$ or -1.

Above constraints ensure that the quench phase shift in Supplementary Eq. (42) is only sensitive to the imaginary part of the response function $\mathrm{Im}G^R_{\xi\xi}[\omega]$, or equivalently the spectral function $\mathcal{J}[\omega]$, so that we have

$$\Phi_{\mathrm{q}}(t_f) = \int_{-\infty}^{+\infty} \mathcal{F}_{\mathcal{J}}[\omega; t_f]\mathcal{J}[\omega]d\omega = 2\int_{0}^{+\infty} \mathcal{F}_{\mathcal{J}}[\omega; t_f]\mathcal{J}[\omega]d\omega, \tag{44}$$

$$\mathcal{F}_{\mathcal{J}}[\omega; t_f] = \frac{\sin^2\frac{\omega t_f}{2}}{4\sin^2\frac{\omega t_f}{2M}}\mathrm{Im}(F_0^*[\omega; 2T]\eta_0[\omega; 2T]). \tag{45}$$

The spectral filter $\mathcal{F}_{\mathcal{J}}[\omega; t_f]$ for $\mathcal{J}[\omega]$ forms a comb-like structure in frequency space, if we fix pulse periodicity $T = t_f/2M$ and take the asymptotic large pulse number limit, i.e.

$$\mathcal{F}_{\mathcal{J}}[\omega; t_f] \sim \frac{M\omega_0}{4}\sum_{\ell=-\infty}^{+\infty} \mathrm{Im}(F_0^*[\ell\omega_0; 2T]\eta_0[\ell\omega_0; 2T])\,\delta(\omega - \ell\omega_0) \quad (M \gg 1), \tag{46a}$$

$$\omega_0 = \pi/T = 2M\pi/t_f. \tag{46b}$$

Thus, given a finite target frequency range, we can construct a corresponding set of NV control pulses that specifically realize frequency comb filters for the spectral function at target frequencies. We can then measure the quench phase shifts under these control pulses in the comb limit (fix $T = t_f/2M$ and choose $M \gg 1$), which in turn enable reconstruction of the spectral function $\mathcal{J}[\omega]$ via Supplementary Eq. (44).

[1] A. Abragam, *The Principles of Nuclear Magnetism*, Comparative Pathobiology - Studies in the Postmodern Theory of Education (Clarendon Press, Oxford, 1961).

[2] C. L. Degen, F. Reinhard, and P. Cappellaro, Quantum sensing, Rev. Mod. Phys. **89**, 035002 (2017).

[3] L. Petit, J. M. Boter, H. G. J. Eenink, G. Droulers, M. L. V. Tagliaferri, R. Li, D. P. Franke, K. J. Singh, J. S. Clarke, R. N. Schouten, V. V. Dobrovitski, L. M. K. Vandersypen, and M. Veldhorst, Spin lifetime and charge noise in hot silicon quantum dot qubits, Phys. Rev. Lett. **121**, 076801 (2018).

[4] J. Bylander, S. Gustavsson, F. Yan, F. Yoshihara, K. Harrabi, G. Fitch, D. G. Cory, Y. Nakamura, J. S. Tsai, and W. D. Oliver, Noise spectroscopy through dynamical decoupling with a superconducting flux qubit, Nat. Phys. **7**, 565 (2011).

[5] T. Rosskopf, A. Dussaux, K. Ohashi, M. Loretz, R. Schirhagl, H. Watanabe, S. Shikata, K. M. Itoh, and C. L. Degen, Investigation of surface magnetic noise by shallow spins in diamond, Phys. Rev. Lett. **112**, 147602 (2014).

[6] E. J. Connors, J. Nelson, and J. M. Nichol, Charge-noise spectroscopy of si/sige quantum dots via dynamically-decoupled exchange oscillations, arXiv preprint arXiv:2103.02448 (2021).

[7] A. A. Clerk, M. H. Devoret, S. M. Girvin, F. Marquardt, and R. J. Schoelkopf, Introduction to quantum noise, measurement, and amplification, Rev. Mod. Phys. **82**, 1155 (2010).

[8] R. J. Schoelkopf, A. A. Clerk, S. M. Girvin, K. W. Lehnert, and M. H. Devoret, Qubits as spectrometers of quantum noise, in *Quantum Noise in Mesoscopic Physics*, edited by Y. V. Nazarov (Springer, Dordrecht, 2003) pp. 175–203.

[9] C. M. Quintana, Y. Chen, D. Sank, A. G. Petukhov, T. C. White, D. Kafri, B. Chiaro, A. Megrant, R. Barends, B. Campbell, Z. Chen, A. Dunsworth, A. G. Fowler, R. Graff, E. Jeffrey, J. Kelly, E. Lucero, J. Y. Mutus, M. Neeley, C. Neill, P. J. J. O'Malley, P. Roushan, A. Shabani, V. N. Smelyanskiy, A. Vainsencher, J. Wenner, H. Neven, and J. M. Martinis, Observation of classical-quantum crossover of $1/f$ flux noise and its paramagnetic temperature dependence, Phys. Rev. Lett. **118**, 057702 (2017).

[10] F. Yan, D. Campbell, P. Krantz, M. Kjaergaard, D. Kim, J. L. Yoder, D. Hover, A. Sears, A. J. Kerman, T. P. Orlando, S. Gustavsson, and W. D. Oliver, Distinguishing coherent and thermal photon noise in a circuit quantum electrodynamical system, Phys. Rev. Lett. **120**, 260504 (2018).

[11] Y.-X. Wang and A. A. Clerk, Spectral characterization of non-gaussian quantum noise: Keldysh approach and application to photon shot noise, Phys. Rev. Research **2**, 033196 (2020).
